# Indirect Evidence Based on Mating-Type Ratios for the Role of Sexual Reproduction in European and Chinese Populations of *Plenodomus biglobosus* (Blackleg of Oilseed Rape)

**DOI:** 10.3390/pathogens12010003

**Published:** 2022-12-20

**Authors:** Kevin M. King, Gail Canning, Kang Zhou, Zekuan Liu, Mingde Wu, Jonathan S. West

**Affiliations:** 1 Rothamsted Research, Harpenden AL5 2JQ, UK; 2 Huazong Agricultural University, Wuhan 430070, China

**Keywords:** blackleg, epidemiology, heterothallic, *Leptosphaeria biglobosa*, mating type, oilseed rape, Phoma, sexual reproduction

## Abstract

Blackleg (Phoma) disease, caused by the ascomycete fungi *Plenodomus biglobosus* and *P. lingam*, threatens oilseed rape (OSR; *Brassica napus*) crops internationally. In many parts of the world, both species co-occur, but in China only *P. biglobosus* has so far been reported. *Plenodomus biglobosus* reproduces asexually (pycnidiospores), but also sexually (pseudothecia-yielding ascospores), via a heterothallic mating system requiring *MAT1-1* and *MAT1-2* genotypes. However, the roles of airborne ascospore inoculum in driving blackleg disease outbreaks in China are less well understood compared to elsewhere in the world. This is despite the very different agronomic cropping practices in parts of China, in which paddy rice and OSR are often grown in rotation; OSR stubble is often submerged under water for long periods potentially affecting pseudothecial development. Here, we indirectly investigate the potential role of sexual reproduction by developing new polymerase chain reaction (PCR) -based mating-type diagnostics for *P. biglobosus* and subsequently screening an international collection of 59 European and 157 Chinese isolates. Overall, in both Europe and China, *P. biglobosus* mating types did not deviate from a 1:1 ratio, such as is generally thought to occur under frequency-dependent selection in sexually reproducing pathogen populations. Both mating types were balanced in all the individual European countries tested (Austria, France, Poland, UK). Conversely, in China, mating types were only balanced in the eastern region; in the northern and southwestern regions there were skewed ratios, more typical of predominantly asexual reproduction, towards *MAT1-1* and *MAT1-2*, respectively. The implications of these findings and future research directions for improved understanding of *P. biglobosus* epidemiology on OSR, particularly in China, are considered.

## 1. Introduction

Blackleg (also Phoma stem canker, Phoma leaf spot) is an internationally important disease of oilseed rape (OSR; *Brassica napus* L.) and other *Brassica* spp., annually causing substantial yield losses in the major growing regions of Australia, Europe and North America [1,2]. Two related fungal pathogen species have been widely identified as the causal agents of the disease in OSR. The first is *Plenodomus lingam*, (Tode:Fr) Desmaz. (formerly *Leptosphaeria maculans* (Desmaz.) Ces. and De Not.), previously referred to as ‘Tox^+^’, ‘A-group’, ‘sirodesmin positive’ and ‘aggressive’. The second species is *Plenodomus biglobosus* (Shoemaker and H. Brun), Gruyter, Aveskamp and Verkley (formerly *Leptosphaeria biglobosa* Shoemaker and H. Brun), and previously referred to as ‘Tox^0^’, ‘B-group’, ‘sirodesmin negative’ and ‘non-aggressive’ [3,4]. Seven genetically distinct *P. biglobosus* ‘subclades’ have so far been identified (subclades ‘americensis’, ‘australensis’, ‘brassicae’, ‘canadensis’, ‘erysimii’, ‘occiaustralensis’ and ‘thlaspii’) [3,5,6], with the ‘brassicae’ and ‘canadensis’ subclades being the most geographically widespread on an international scale and the only subclades detected so far on the *Brassica* species in Europe and China [3,7,8,9]. While *P. biglobosus* and *P. lingam* are by far the more internationally important Phoma pathogens in OSR [2], a third species named *Plenodomus dezfulensis,* Mehrabi-Koushki, Safi and Farokhinejad, has also very recently been described in OSR [10] that is more closely related to *P. biglobosus*, but this new species has so far been isolated very infrequently from only a few very diseased leaves in Iran. Lastly, an additional species, *Plenodomus wasabiae*, Yokogi ex J.F. White and P.V. Reddy (basionym *Phoma wasabiae* Yokogi), has also been reported on wasabi (*Eutrema japonicum* (Miq.) Koidz.), but ITS sequence analyses indicate that this species does in fact share 100% identity with the *P. biglobosus* subclade ‘occiaustralensis’ [4,11]. In this study, for clarity, the names *P. biglobosus* (and its constituent seven genetic subclades) and *P. lingam* will be used [4], along with the recently described species *P. dezfulensis* [10] that will also occasionally be referred to.

Both *P. biglobosus* and *P. lingam* are currently known to co-occur in many parts of the world, including in OSR crops in Australia, Canada and Europe [2]. However, to date, only *P. biglobosus* has so far been identified in China and there are concerns that accidental introduction and spread of *P. lingam* in China might in future occur and potentially threaten the ca. 7M Ha of winter OSR grown in central and southern China and the ca. 1M Ha of spring OSR grown in northern China [12]. *Plenodomus lingam* is often associated with more damaging lower stem (crown cankers) lesions while *P. biglobosus* is more often linked to the less damaging upper stem lesions [2], although there are data to suggest that both species can be obtained from upper and basal stem lesions [13] and there is evidence that *P. biglobosus* can sometimes cause considerable yield losses in OSR crops including in both Europe and China [7,8,14,15,16].

Blackleg pathogen inocula are known to reproduce via asexually produced splash-dispersed pycnidiospores and/or sexually derived airborne ascospores, with the relative importance of each appearing to vary by geographical location such as in Europe (mostly ascospores), Australia (mainly ascospores, also pycnidiospores) and western Canada (mostly pycnidiospores) [13,17,18]. The relative importance of each inoculum type remains unclear in some other parts of the world, including China. Studies investigating the patterns of *P. lingam* and *P. biglobosus* ascospore release, by applying species-specific quantitative polymerase chain reaction (qPCR) assays to air samples, have so far been conducted mostly in Europe [19,20,21,22]. Similar work is now required to investigate the potential role of airborne ascospore production of each of the species in blackleg epidemics in other countries in which blackleg is problematic. Both *P. lingam* and *P. biglobosus* are known to have heterothallic sexual cycles yielding pseudothecia and ascospores, requiring both *MAT1-1* and *MAT1-2* genotypes in order to occur. In sexually reproducing pathogen populations, 1:1 ratios of such mating types are typical under such frequency-dependent selection [23], and thus mating-type ratios can potentially be useful as an indirect measure to infer the importance of sexual reproduction. A mating-type-specific multiplex PCR diagnostic for *P. lingam* was developed by Cozijnsen and Howlett (2003) [24], but this assay does not work for isolates of *P. biglobosus* [6]. The use of this diagnostic has shown balanced mating-type ratios in some *P. lingam* populations from Australia [25], France [26] and Idaho in the US [27], but not in others including Mexico [28] and New Zealand [29]. In contrast, for *P. biglobosus*, only one molecular study investigating mating type has so far been conducted. Voight et al. (2005) [30] screened 18 international *P. biglobosus* isolates and a fragment of the *MAT1-2* locus was amplified from 12, while additional PCR testing amplified a fragment of what may have been from the *MAT1-1* locus from an additional 2. An examination of mating-type distributions of *P. biglobosus* isolates on an international scale is now required to indirectly investigate the role of sexual reproduction in different geographic regions, especially in parts of the world where only this species is known to occur, such as China [7,8,16].

The main aim of this study is to improve blackleg disease management through better understanding of *P. biglobosus* population genetics and epidemiology. Thus, we here describe the development of new mating-type diagnostics capable of discriminating between *MAT1-1* and *MAT1-2* isolates of *P. biglobosus.* We then apply these new diagnostics to explore mating-type ratios in *P. biglobosus* populations from Europe and China to indirectly investigate the role of sexual reproduction in these pathogen populations.

## 2. Materials and Methods

### 2.1. Fungal Isolates and DNA Extraction

An international collection of *P. biglobosus* isolates from across Europe and China was assembled from two sources. The first, from the Oilseed Rape Enhanced Genetic Improvement Network (OREGIN; www.herts.ac.uk/oregin; accessed on 7 December 2022 [31]), comprised 109 *P. biglobosus* isolates in total, with 50 from China (all from OSR stems; 2 collected in 1999; 48 collected in 2005/2006) and a further 59 from Europe (all from OSR; mostly from stems but occasionally leaf lesions; collected over many years). The identity of all these isolates had previously been confirmed as *P. biglobosus* using species-specific PCR diagnostics [7]. The second, the Huazong culture collection, comprised 107 *P. biglobosus* isolates (species identities again confirmed using species-specific PCR) in total, collected from OSR and *B. rapa* between 2018/2019. Thus, in total, 216 *P. biglobosus* isolates were used in the present study, with details as described in Table 1. DNA extraction from the OREGIN collection was as described in Liu et al. (2014) [7], after which genomic DNA was quantified by nanodrop and diluted to 10 ng/μL.

### 2.2. Design of New Plenodomus biglobosus Mating-Type PCR Diagnostics

Attempts to apply the multiplex mating-type diagnostic assay, Cozijnsen and Howlett (2003), designed for closely related *P. lingam* to a range of isolates of *P. biglobosus* were unsuccessful [24]. New *MAT1-1*-specific PCR primers were designed based on *MAT1-1* idiomorph sequence data downloaded from the available *P. biglobosus* genome (European Nucleotide Archive (ENA): PRJEB24467; [32]); primers PbMAT1F (5′ TGAAGTTGGCCGCTTCCTC 3′) and PbMAT1R (5′ CTGAGGCGGCATTGGGGTC 3′) were targeted to a 640bp fragment of the *MAT1-1* idiomorph. New *MAT1-2*-specific primers for *P. biglobosus* were targeted to *MAT1-2* idiomorph sequence and were designed based on sequence data retrieved from GenBank (AY748930, AY748939); primers PbMAT2F (5′ GAGTCGGAGAAGAAGCCCTG 3′) and PbMAT2R (5′ ATTCCGGCTTCGAACTCCTC 3′) were targeted to a 416bp fragment of the *MAT1-2* idiomorph.

### 2.3. Validation of New Plenodomus biglobosus PCR Mating-Type Diagnostics

Seven *Plenodumus* spp. isolates were used for initial validation of the new mating-type diagnostics designed in this study. These included two isolates of *P. biglobosus* ‘brassicae’ (KO8 from Iran; 21WAS8-4 from the UK) and two isolates of *P. biglobosus* ‘canadensis’ (21WAS1-2 and 21WAS7-1, both from the UK); isolate, species and subclade identities had previously been confirmed based on at least ITS sequences [9,33]. An isolate of the closely related *P. dezfulensis* (SCUA-Ahm-S41, Iran) [10] was also tested. Furthermore, two *P. lingam* isolates were tested from the OREGIN collection that had previously been molecularly characterised, using the *P. lingam* mating-type diagnostic of Cozijnsen and Howlett (2003) [24], as either a *MAT1-1* (IBCN80, from Canada) or *MAT1-2* (WAC4057, from Australia) genotype.

Each PCR was performed in 12.5 μL volume containing 0.1 μL each of forward and reverse primer (each at 0.16 μM final reaction concentration), 6.25 μL of 2× RedTaq ReadyMix (1× final concentration; Sigma-Aldrich, UK), 5.05 μL of PCR grade water and 1 μL of genomic DNA (10 ng total). Reaction conditions were 35 cycles of 94 °C for 1 min, either 62 °C (for primer pair PbMAT1F/R) or 60 °C (for primer pair PbMAT2F/R) for 1 min and 72 °C for 1 min, with a final extension of 72 °C for 5 min. PCR amplicons (8 μL) were resolved on 2% agarose gels containing GelRed and viewed under UV light. PCR amplicon sizes were subsequently purified using a MinElute PCR purification kit (Qiagen, Germany) and sent to MWG Eurofin (Germany) for bidirectional sequencing.

### 2.4. PCR Testing of European and Chinese Plenodomus biglobosus Isolates for Mating Type

Two hundred and sixteen *P. biglobosus* isolates from Europe and China were subsequently screened for mating type using the newly developed diagnostic assays as previously described (Table 1). The hypotheses of 1:1 ratios of mating types (within individual countries/regions and overall) were tested statistically using a *X*^2^ test (GraphPad Prism 9 Software, San Diego, CA, USA).

## 3. Results

### 3.1. Validation of New Plenodomus biglobosus Mating-Type Diagnostics

**Figure 1:**
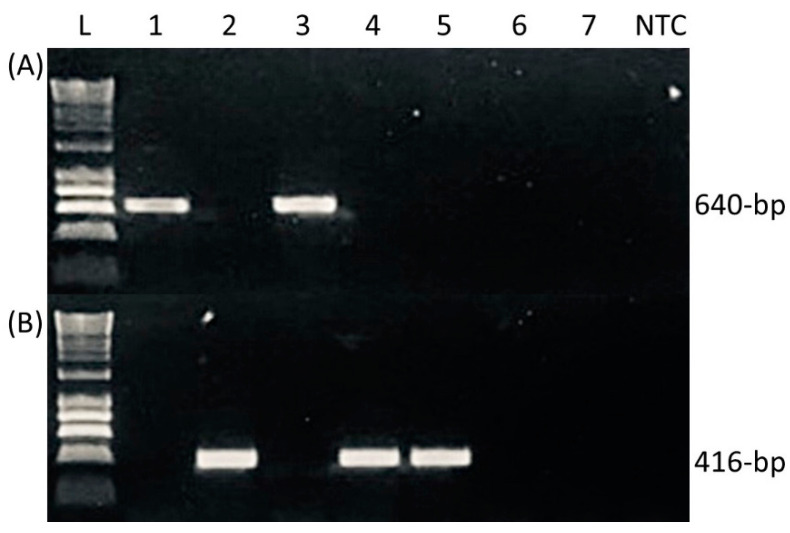
Validation of new mating-type polymerase chain reaction (PCR) diagnostics designed to discriminate between (**A**) *MAT1-1* (primers PbMAT1F/R, amplicon 640bp) and (**B**) *MAT1-2* (primers PbMAT2F/R, amplicon 416bp) isolates of *Plenodomus biglobosus*. Isolates screened in lanes are 1: KO8 (*P. biglobosus* ‘brassicae’, *MAT1-1*); 2: 21WAS8-4 (*P. biglobosus* ‘brassicae’, *MAT1-2*); 3: 21WAS1-2 (*P. biglobosus* ‘canadensis’, *MAT1-1*); 4: 21WAS7-1 (*P. biglobosus* ‘canadensis’, *MAT1-2*); 5: SCUA-Ahm-S41 (*P. dezfulensis*, *MAT1-2*); 6: Leroy (*P. lingam*, *MAT1-1* type, no amplicon); 7: WAC 4057 (*P. lingam*, *MAT1-2* type, no amplicon); 8: no template water control (NTC).

Initial PCR testing showed that putative *MAT1-1* amplicons of the expected ~640 bp size were successfully amplified using primers PbMAT1F/R from the *P. biglobosus* isolates KO8 (subclade ‘brassicae’) and 21WAS1-2 (subclade ‘canadensis’) (Figure 1A). No amplicons were obtained for either of the *P. lingam* isolates tested (including the *MAT1-1* isolate IBCN80) nor for the *P. dezfulensis* isolate SCUA-Ahm-S41. Bidirectional sequencing of isolates KO8 and 21WAS1-2 yielded partial sequences of 572bp; BLAST analyses confirmed the closest GenBank hits (75.7–76.5% identity) to be a region of the *MAT1-1* gene of *P. lingam* (Accession AY174048). The *MAT1-1* gene sequences obtained in this study for isolates KO8 and 21WAS1-2 shared 97.9% identity with each other, and have been deposited onto GenBank (Accessions OP555983 and OP555984, respectively).

Further PCR testing revealed that putative *MAT1-2* products of the expected ~416 bp size were, however, instead successfully amplified with primers PbMAT2F/R from *P. biglobosus* isolates 21WAS8-4 (subclade ‘brassicae’) and 21WAS7-1 (subclade ‘canadensis’) and also from the *P. dezfulensis* isolate SCUA-Ahm-S41 (Figure 1B). No amplicons were obtained for either of the *P. lingam* isolates tested (including the *MAT1-2* isolate WAC4057). Bidirectional sequencing of the amplicons yielded partial sequences of 382bp. BLAST analyses showed that sequences obtained from the *P. biglobosus* isolates 21WAS8-4 and 21WAS7-1 showed 100% identities to GenBank *MAT1-2* gene sequences of the *P. biglobosus* subclades ‘brassicae’ (Accession AY748934) or ‘canadensis’ (Accession AY748940), respectively. The *MAT1-2* gene sequences newly obtained in this study for isolates 21WAS8-4 and 21WAS7-1 showed 95.8% identity and have been deposited onto GenBank (Accessions OP555985 and OP555986, respectively). Lastly, the sequence obtained from *P. dezfulensis* isolate SCUA-Ahm-S41 shared a higher identity with the partial *MAT1-2* sequence of the *P. biglobosus* isolate 21WAS7-1 (97.4%) than isolate 21WAS8-4 (96.3%). The partial *MAT1-2* gene sequence of the *P. dezfulensis* isolate SCUA-Ahm-S41 has been deposited onto GenBank (Accession OP555987).

### 3.2. PCR Screening of European and Chinese P. biglobosus Isolates for Mating Type

All 216 of the European and Chinese *P. biglobosus* isolates tested could be assigned to either the *MAT1-1* or *MAT1-2* genotype (Table 1). Screening with primers PbMAT1R/F yielded the expected ~640 bp amplicon for *MAT1-1* isolates, whereas the use of primers PbMAT2F/R gave the predicted ~416 bp product for *MAT1-2* isolates. An additional six Canadian *P. biglobosus* isolates were also tested and were identified as either a *MAT1-1* or *MAT1-2* genotype, demonstrating the robustness of the developed diagnostics to *P. biglobosus* isolates from other geographic regions.

For the 59 European isolates tested, no statistically significant departure from a 1:1 ratio of *MAT1-1*:*MAT1-2* types was found, such being the case for each of the individual French, Polish and UK populations screened. Five Austrian isolates were also tested, with both *MAT* types again present, but this sample could not be analysed statistically due to small sample numbers. For the remaining 157 Chinese isolates tested, when all data were combined, no evidence was obtained for a departure from a 1:1 ratio of *MAT1-1*:*MAT1-2* types. However, it should be noted that upon inspection of underlying data supporting this headline result, two of the three Chinese regions had ratios that were significantly different from a 1:1 mating-type ratio (*p <* 0.01). Thus, although the eastern region had a balanced mating-type ratio, the northern and southwestern regions were heavily skewed towards *MAT1-1* and *MAT1-2* type isolates, respectively.

## 4. Discussion

In this study, new PCR-based diagnostics were successfully developed to discriminate between *MAT1-1* and *MAT1-2* genotypes of *P. biglobosus*. The diagnostics were validated by screening against *P. biglobosus* subclades ‘brassicae’ and ‘canadensis’, the two most geographically widespread subclades on an international scale, and the only ones reported to date on *Brassica* species in Europe and China. The wider applicability of the new *MAT1-2* diagnostic was further demonstrated by its successful application to *P. dezfulensis*, a recently described species that is very closely related to the *P. biglobosus* subclades [10]. It is noted that the new *P. biglobosus* mating-type diagnostics described here do not work for the more distantly related *P. lingam*, but the latter species’ mating-type diagnostics were previously developed by Cozijnsen and Howlett (2003) [24].

The new *P. biglobosus* mating-type diagnostics were subsequently applied to European and Chinese isolates of this species. For the China dataset, no evidence for deviation from a 1:1 mating-type ratio was found in combined data from the three geographic regions tested (east, north, southwest). Similar results supporting a 1:1 ratio were also obtained in the European dataset, based on combined mating-type data from four countries (Austria, France, Poland, UK). Thus, these combined data provide indirect evidence for the role of sexual reproduction in *P. biglobosus* in Europe, as previously supported by air spore trapping studies [19,20,21,22], and also in China, although there such air trapping studies do not to the authors’ knowledge appear to have been conducted. This is also consistent with the genetic diversity previously detected in European and Chinese *P. biglobosus* populations [7,8], a feature consistent with regular sexual reproduction [23].

Despite this overall result, however, closer inspection of the Chinese mating-type dataset revealed that, of the three individual regions examined, only the eastern region exhibited a 1:1 distribution. In contrast, skewed distributions that deviated significantly from a 1:1 ratio were observed in both the northern region (91% *MAT1-1*) and southwest region (64% *MAT1-2*). In contrast, for the European mating-type dataset, no evidence for a departure from a 1:1 distribution was obtained in any of the individual countries tested. Skewed mating-type distributions, such as those observed in the northern and southwestern regions of China in this study, are more typical of asexual (clonally) reproducing populations. Previous work by West et al. (2000) [34] investigated sexual reproduction using blackleg-diseased OSR stubble (that was incubated for three months under natural conditions), collected from three different sites in China. Sexual reproduction, with pseudothecia-yielding ascospores, was confirmed from stubble collected from Guizhou (southwestern region) and Anhui (eastern region), but not from Hubei (southwestern region). One hypothesis is that the stubble of West et al. (2000) [34] collected from Hubei contained only a single mating type, thus precluding completion of the sexual cycle.

Further study is now required to explore the extent to which sexually produced ascospores drive blackleg outbreaks in China, especially since the relative importance of ascospore inoculum appears to vary between other major OSR-growing regions (Australia, Canada and Europe) of the world [1,17,18]. Although combined mating-type data from throughout China obtained in this study overall provide indirect evidence for the role of sexual cycles there, data from individual regions suggest that asexual reproduction may predominate in some locations. Although it is acknowledged that the *P. biglobosus* isolates used in this study were not sampled in a systematic way, nor were the resulting dataset’s clones corrected, many of the European and Chinese OREGIN collection isolates tested here had previously been analysed via AFLP and found to be genetically distinct [7]. More comprehensive investigations, using the *P. biglobosus* mating-type-specific diagnostics developed here, are now required to investigate mating-type distributions at different spatial scales (i.e., continent, region, field, leaf, lesion), and such work should incorporate these factors into their design.

Future work should additionally focus on the quantification of *P. biglobosus* airborne inocula at different locations in China through air spore trapping in conjunction with species-specific qPCR. As previously discussed, such studies have been conducted in Europe but not, to the authors’ knowledge, yet in China. It is possible that sexual reproduction may occur less frequently in some Chinese *P. biglobosus* populations compared to other populations in the world, due to the major importance of OSR and flooded paddy rice (*Oryza sativa*) crop rotations there. This is because in such agricultural systems, blackleg-diseased crop debris is often submerged under water for extended periods of time, and there is evidence that the flooding of infected debris for even relatively short time periods (10 days) may inhibit ascospore production and release [35]. Understanding the extent to which sexual reproduction might be occurring in the field for Chinese *P. biglobosus* populations could provide novel insights, for instance into inoculum dispersal and the evolutionary potential of the population there [23], that could be used to inform blackleg disease management strategies.

## Figures and Tables

**Table 1:** Mating-type distribution as determined via polymerase chain reaction (PCR) of 216 Chinese and European *Plenodomus biglobosus* isolates.

Isolates Origin	Total Isolates Tested ^a^	*MAT1-1*:*MAT1-2* Frequency	*X*^2^ Value	*p* Value
**China:**				
North (Inner Mongolia, Shaanxi)	22 (OSR: 22)	20:2	14.72	<0.01
East (Anhui, Jiangsu)	46 (OSR: 18; BR: 28)	23:23	0	1.00
Southwest (Chongqing, Guizhou, Hubei, Hunan, Sichuan)	89 (OSR: 56; BR: 33)	32:57	7.02	<0.01
Grand total China:	157 (OSR: 96; BR: 61)	75:82	0.31	0.576
**Europe:**				
Austria	5 (OSR: 5)	3:2	-	-
France	13 (OSR: 13)	5:8	0.69	0.405
Poland	15 (OSR: 15)	5:10	1.67	0.197
United Kingdom	26 (OSR: 26)	15:11	0.62	0.433
Grand total Europe:	59 (OSR: 59)	28:31	0.15	0.691
**China + Europe datasets combined:**				
Grand total (China + Europe)	216 (OSR: 155; BR: 61)	103:113	0.46	0.496

^a^ Number of isolates collected from *Brassica napus* (oilseed rape, OSR) or *Brassica rapa* (BR) indicated in brackets.

## Data Availability

The data that support the findings of this study are available from the corresponding author (K.M.K.) upon reasonable request.
